# Association between dietary folate intake and severe headache among adults in the USA: a cross-sectional survey

**DOI:** 10.1017/S000711452300137X

**Published:** 2024-02-14

**Authors:** Sheng Tian, Lanxiang Wu, Heqing Zheng, Xianhui Zhong, Mingxu Liu, Xinping Yu, Wei Wu

**Affiliations:** Department of Neurology, The Second Affiliated Hospital of Nanchang University, Nanchang, Jiangxi 330006, People’s Republic of China

**Keywords:** NHANES, Folate, Severe headache, Cross-sectional study, Restricted cubic spline

## Abstract

Folate, also known as vitamin B_9_, is a water-soluble vitamin. Previous studies on dietary folate intake in severe headache patients were equivocal. Therefore, we conducted a cross-sectional study to elucidate the relationship between folate intake and severe headache. This cross-sectional study used data from participants over 20 years old who participated in the National Health and Nutrition Examination Survey (NHANES) from 1999 to 2004. The diagnosis of severe headache was made through participants’ self-report in the NHANES questionnaire section. We performed multivariate logistic regression and restricted cubic spline (RCS) regression to explore the relationship between folate intake and severe headache. A total of 9859 participants took part in the study, 1965 of whom were severe headache patients and the rest were non-severe headache. We found that dietary folate intake was significantly and inversely associated with severe headache. Compared with participants with lower folate intake Q1 (≤ 229·97 ug/d), the adjusted OR values for dietary folate intake and severe headache in Q2 (229·98–337 ug/d), Q3 (337·01–485 ug/d) and Q4 (≥ 485·01 ug/d) were 0·81 (95 % CI: 0·67, 0·98, *P* = 0·03), 0·93 (95 % CI: 0·77, 1·12, *P* = 0·41) and 0·63 (95 % CI: 0·49, 0·80, *P* < 0·001), respectively. For women aged 20–50 years, there was a non-linear association between folate intake and severe headache in the RCS. Women aged 20–50 years should have higher awareness of dietary folate and increase their dietary intake of folate, which may aid in preventing severe headache.

Severe headache or migraine is a common headache disorder with a high prevalence in people less than 50 years old, causing severe disability to the individual and a major burden to society^(1)^. It is characterised by paroxysmal attacks with headache, nausea and increased sensitivity to movement, light and sound^(2)^. The American Migraine Prevalence and Prevention Study indicated that the majority of people reported to have ‘severe headaches’ met the diagnostic criteria for migraine or possible migraine^(3)^.

There are two main subtypes of migraine, including migraine with aura and migraine without aura. These two entities can be distinguished by focal neurological symptoms that typically precede or accompany headache attacks in patients having migraine with aura^(4)^. Cortical spreading depression is a phenomenon of spreading depolarisation across the cerebral cortex, which is related to the presence of aura^(5)^. Among the genetic and environmental factors that may contribute to the development of migraine, a study has shown a possible link between 5,10-methylenetetrahydrofolate reductase gene polymorphisms associated with reduced enzyme activity and migraine risk^(6)^. Defective or insufficient production of methylenetetrahydrofolate reductase, an important enzyme involved in the metabolism of homocysteine (Hcy), can lead to hyperhomocysteinaemia^(7)^. Rainero I et al suggested that interventions targeting Hcy metabolism, primarily supplementation with Hcy-lowering vitamins (including folate), may have beneficial effects on severe headache or migraine patients^(8)^.

Folate is a water-soluble vitamin and its synthetic form is folic acid^(9)^. The metabolism of Hcy is reported to be dependent on the presence of the cofactor folate, and in the presence of folate deficiency, Hcy levels tend to be elevated^(10)^. Smith AD et al revealed that there was a correlation between the level of serum Hcy and the frequency and characteristics of headache attacks^(11)^. And dietary compounds, folate, was found to reduce Hcy levels in many pathologies^(12)^. A previous study showed that a significant relieving in headache severity, frequency and duration of headache attacks in ninety-five migraine patients with a diet supplementation with pyridoxine and folate for 3 months^(13)^. However, the relationship between dietary folate intake and severe headache has not been reported in the general population.

The relationship between dietary folate intake and severe headache in adults was examined using data from the National Health and Nutrition Examination Survey to refine this study in the general population. Based on the nutritional patterns found in this population, we hypothesised that dietary folate consumption would be inversely associated with severe headache. Furthermore, a dose–response relationship between dietary folate intake and severe headache was descripted.

## Materials and methods

### Study population

Data analysed in this cross-sectional study was obtained from NHANES, administered by the Centers for Disease Control and Prevention. The NHANES data is a series of cross-sectional, stratified, multi-stage probability surveys for the Americans, non-institutional population of the USA^(14)^. The NHANES collects information on demographics, laboratory tests, physical examinations, diet surveys and other health-related questions via home visits and mobile examination center. All NHANES protocols were authorised by the National Center for Health Statistics Ethics Review Committee and the NHANES obtained written informed consent signed by all participants. No additional institutional review board approval was required for the secondary analysis^(15)^. Relevant data from NHANES are publicly available, and its methodological details and survey design can be found at https://www.cdc.gov/nchs/nhanes/index.htm.

**Fig. 1.**
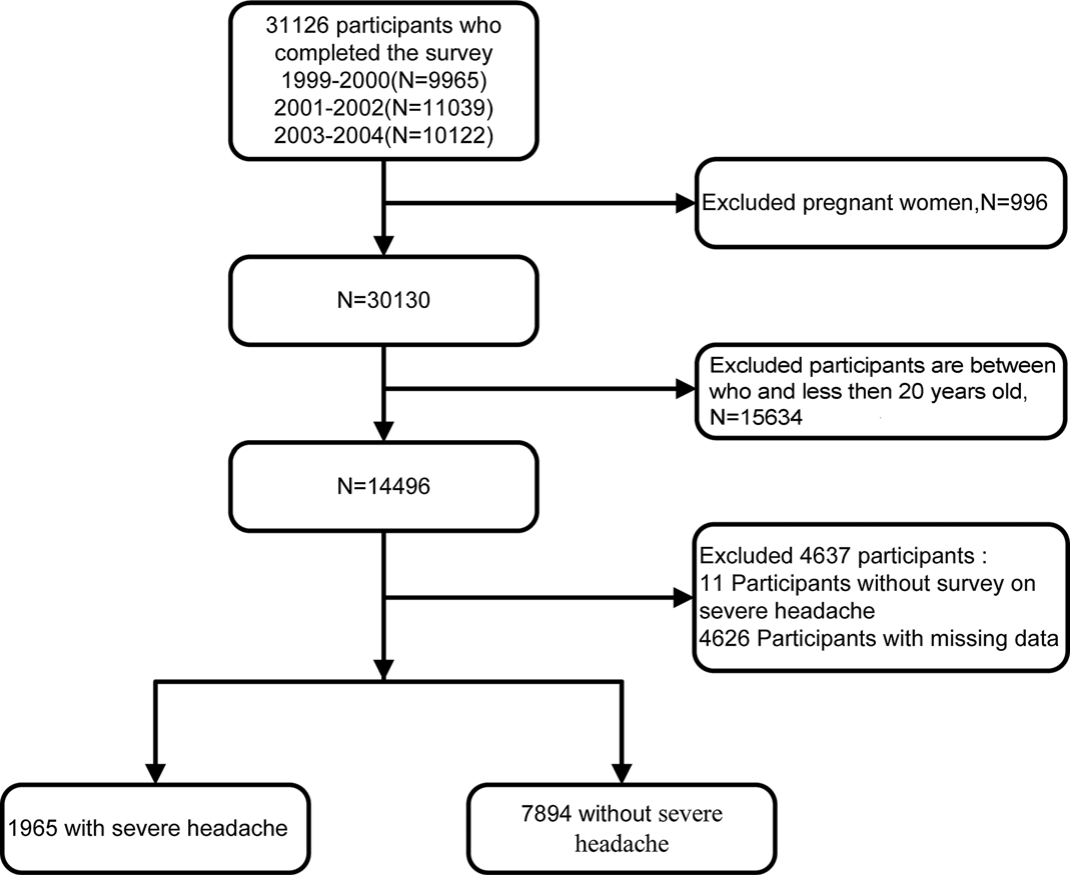
Flow chart of participants inclusion and exclusion for analysis.

The data were not subjected to statistical weight calculations prior to the study, and the sample size was based on all available data in NHANES. We conducted a cross-sectional study of American adults from the 1999–2004 NHANES survey, as this was the only cycle to include headache questionnaires for adults. The data were combined for our analysis, resulting in 31 126 participants, and our study was limited to adults 20 years old or older. We excluded participants without information such as severe headache, pregnant women and missing data. Finally, 9859 subjects remained in our study (Fig. 1).

### Severe headache classification

We evaluated severe headache by self-report in the NHANES questionnaire section. We divided participants who answered ‘yes’ into those with severe headache: ‘In the past 3 months, have you had severe headaches or migraines?’ We may consider that the majority of participants with severe headaches have migraine. Our findings are consistent with those of the American Migraine Prevalence and Prevention study. The study revealed that 17·4 % of participants reported ‘severe headache’, of which 11·8 % met the International Headache Disorder Type II criteria for migraine, 4·6 % met the criteria for ‘probable migraine’ and only 1 % were identified as ‘other severe headache’^(3)^.

### Dietary folate assessment

Dietary folate intake was identified through a 24-h recall survey. The survey is a retrospective dietary evaluation method that provides detailed information on all meals and beverages consumed over a 24-h period^(16)^. The dietary assessments were conducted in person by trained dietary investigators. Participants were shown a standard set of measurement guidelines that help them to be able to accurately report the quantity and size of food. NHANES codes the acquired data by using the Food Intake Analysis System and the USA Department of Agriculture survey nutrition database, which in turn converts them into total nutrient intakes^(17)^. In the present study, all participants performed their first 24-h dietary recall from 1999 to 2004^(18)^. Furthermore, we grouped each participant by their dietary folate intake.

### Potential covariates

Various potential covariates were assessed based on previous literature^(18–20)^. Demographic covariates included age, gender, ethnicity, education level, marital status and family income, all of which were obtained through self-expression during the interview process. Lifestyle-related covariates included smoking status, alcohol consumption and BMI. Dietary-related covariates included energy consumption, protein consumption, carbohydrate consumption and fat consumption. Clinically relevant covariates included hypertension, diabetes, stroke and coronary heart disease and C-reactive protein.

### Statistical analyses

We performed statistical analysis by using R software (version 4.2.1). All analyses in this study were based on appropriate sampling weight to account for the complex survey design^(21)^. The statistical descriptions of continuous variables were presented as sample-weighted means (standard errors), whereas categorical variables are reported as sample-weighted percentages and frequencies. To compare the differences between different groups, one-way analyses of variance was performed for continuous variables, but *χ* ^2^ test was performed for categorical variables. Also, we divided the participants into quartile by dietary folate consumption. Multifactorial logistic regression was used to examine the association between dietary folate consumption and severe headache, and relevant data were expressed as OR (95 % CI). To assess the robustness of the results, a sensitivity analysis was also conducted on participants without extreme energy intake (consuming < 500 or > 5000 kcal per day) for sensitivity analyses. In addition, restricted cubic spline regression was used to explore the non-linear relationship between dietary folate and severe headache. And dietary folate consumption was involved in the model as a continuous variable. Two-sided *P* values < 0·05 were considered statistically significant difference.

## Results

### Baseline characteristic

The basic characteristics of the included and excluded individuals are exhibited in the Supplementary Materials (Table S1). Table 1 shows a total of 1965 individuals with severe headache. The average age of the individuals in this study was 46·10(0·30) years, and 4852(50·65) individuals were female. Participants who consumed more folate often tended to be younger, male, married, non-Hispanic White, had a higher educational level, had a high family income, never smoking never drinking, had a lower incidence of hypertension, diabetes and stroke, had higher consumption of energy, proteins, fat and carbohydrates and lower serum C-reactive protein levels and BMI. As shown in Table 2, the results of the univariate analysis indicated that age, sex, race, education, family income, smoking status, drinking status, coronary heart disease, BMI, protein intake and C-reactive protein were associated with severe headache.

**Table 1.** Population characteristics by categories of dietary folate intake

Characteristic	Folate intake, ug/d										
			Q1		Q2		Q3		Q4		
	Total		≤ 229·97		229·98–337		337·01–485		≥ 485·01		*P* value
	Mean	se	Mean	se	Mean	se	Mean	se	Mean	se	
No.	9859		2465		2467		2465		2462		
Age (years)	46·10	0·30	47·28	0·42	46·54	0·56	46·83	0·43	43·95	0·42	< 0·0001
Sex											< 0·0001
Male	5007	49·35	912	33·25	1119	43·82	1318	51·55	1658	66·43	
Female	4852	50·65	1553	66·75	1348	56·18	1147	48·45	804	33·57	
Marital status											0·003
Living alone	3675	34·47	1075	38·89	937	35·28	842	31·90	821	32·36	
Married	6184	65·53	1390	61·11	1530	64·72	1623	68·10	1641	67·64	
Race											< 0·0001
Non-Hispanic White	5226	73·94	1165	70·60	1281	72·30	1403	76·39	1377	76·01	
Non-Hispanic Black	1792	9·72	624	14·36	486	10·72	344	7·18	338	7·21	
Mexican American	2115	6·78	510	6·26	510	6·28	534	6·74	561	7·74	
Others	726	9·56	166	8·78	190	10·70	184	9·69	186	7·74	
Education level											< 0·0001
< High school	2978	18·53	925	24·57	767	19·35	660	16·13	626	14·84	
High school	2354	26·15	614	28·64	592	26·48	604	26·41	544	23·40	
> High school	4527	55·32	926	46·79	1108	54·17	1201	57·47	1292	61·76	
Family income											< 0·0001
Low	2684	20·55	868	28·87	671	20·52	575	17·58	570	16·21	
Medium	3832	36·02	942	36·62	991	37·99	990	36·60	909	33·08	
High	3343	43·43	655	34·51	805	41·49	900	45·82	983	50·70	
Smoking status											< 0·0001
Never	4944	49·84	1215	47·58	1234	49·56	1241	50·18	1254	51·77	
Current	2201	24·90	644	31·91	542	24·77	519	22·95	496	20·81	
Former	2714	25·25	606	20·52	691	25·67	705	26·87	712	27·42	
Drinking											< 0·0001
Never	1395	12·22	435	14·70	345	12·25	337	11·96	278	10·29	
Current	6421	70·72	1426	65·23	1567	69·55	1658	71·81	1770	75·54	
Former	2043	17·06	604	20·07	555	18·20	470	16·23	414	14·17	
Diabetes	996	6·71	272	7·03	280	7·47	224	6·49	220	5·95	0·044
Hypertension	3265	27·67	942	32·43	847	27·88	806	28·09	670	22·89	< 0·0001
Stroke	322	2·29	114	3·28	88	2·31	68	2·06	52	1·63	0·002
Coronary heart disease	475	3·69	131	3·99	124	4·19	116	3·53	104	3·13	0·27
BMI (kg/m^2^)	28·13	0·11	28·66	0·14	28·11	0·12	28·22	0·16	27·59	0·19	< 0·001
Energy (kcal/d)	2233·75	13·77	1434·31	16·61	1996·78	17·56	2361·18	21·78	3029·46	33·15	< 0·0001
Protein intake (g/d)	82·94	0·68	53·92	0·78	73·93	0·91	87·67	0·99	112·11	1·33	< 0·0001
Carbohydrate intake(g/d)	273·37	1·85	173·44	2·46	239·35	2·62	285·36	2·82	380·82	4·49	< 0·0001
C-reactive protein (mg/dl)	0·41	0·01	0·51	0·02	0·42	0·01	0·40	0·02	0·34	0·02	< 0·0001
Fat consumption (g/d)	84·12	0·58	55·25	0·74	77·34	0·98	89·79	1·31	110·14	1·40	< 0·0001
Severe headache	1963	21·66	451	26·35	390	21·32	382	22·68	371	16·87	< 0·0001

Notes: Continuous variables were shown as mean ± se, and *P* value was calculated by weighted one-way analyses of variance. Categories variables were shown as percentage, and *P* value was calculated by weighted *χ* ^2^ test.

**Table 2.** Relationship of covariates and severe headache risk

Variable	OR	95 % CI	*P* value
Age (years)	0·98	0·98, 0·98	< 0·0001
Sex			
Female	1 (Reference)		
Male	0·49	0·43, 0·55	< 0·0001
Marital status			
Living alone	1 (Reference)		
Married	0·95	0·85, 1·06	0·33
Race			
Non-Hispanic White	1 (Reference)		
Non-Hispanic Black	1·26	1·05, 1·51	0·012
Mexican American	1·17	0·99, 1·38	0·064
Others	1·28	0·96, 1·72	0·09
Education level			
< High school	1 (Reference)		
High school	0·84	0·73, 0·97	0·02
> High school	0·66	0·56, 0·78	< 0·0001
Family income			
Low	1 (Reference)		
Medium	0·68	0·57, 0·82	< 0·001
High	0·47	0·39, 0·56	< 0·0001
Smoking status			
Never	1 (Reference)		
Current	1·35	1·15, 1·57	< 0·001
Former	0·75	0·64, 0·88	< 0·001
Drinking			
Never	1 (Reference)		
Current	0·82	0·71, 0·96	0·02
Former	1·05	0·84, 1·31	0·66
Diabetes			
No	1 (Reference)		
Yes	0·91	0·77, 1·09	0·31
Hypertension			
No	1 (Reference)		
Yes	1·07	0·96, 1·18	0·21
Stroke			
No	1 (Reference)		
Yes	1·43	0·99, 2·05	0·05
Coronary heart disease			
No	1 (Reference)		
Yes	0·73	0·48, 0·99	0·04
BMI (kg/m^2^)	1·02	1·01, 1·03	< 0·0001
Energy (kcal/d)	0·98	0·99, 1·23	0·1112
Protein intake (g/d)	0·99	0·98, 0·99	< 0·001
Carbohydrate intake(g/d)	1·00	0·99, 1·01	0·24
C-reactive protein(mg/dl)	1·10	1·04, 1·17	0·0014
Fat consumption (g/d)	0·99	0·99·02	0·13

### Association between dietary folate consumption and severe headache

The results of the multi-factor logistic regression models are descripted in Table 3. Crude was the unadjusted model, and model 1 was adjusted for age and gender. In addition, model 2 was adjusted for age, gender, race, marital status, education level, family income, smoking status, drinking status, hypertension, diabetes, stroke, coronary heart disease, BMI, energy consumption, protein consumption, carbohydrate consumption, fat consumption and C-reactive protein.

**Table 3.** Association between dietary folate intake and severe headache

Quartiles		Crude			Model 1			Model 2		
	No.	OR	95 % CI	*P* value	OR	95 % CI	*P* value	OR	95 % CI	*P* value
Dietary folate (ug/d)										
Q1(≤ 229·97)	2465	1·00 (reference)			1·00 (reference)			1·00 (reference)		
Q2(229·98–337)	2467	0·76	0·64, 0·90	0·002	0·79	0·66, 0·94	0·01	0·81	0·67, 0·98	0·03
Q3(337·01–485)	2465	0·82	0·70, 0·96	0·013	0·92	0·78, 1·08	0·29	0·93	0·77, 1·12	0·41
Q4(≥ 485·01)	2462	0·57	0·49, 0·66	< 0·0001	0·66	0·56, 0·78	< 0·0001	0·63	0·49, 0·80	< 0·001
*P* trend	–	< 0·0001		–	< 0·0001		–	0·001		–

Crude was adjusted for nothing; model 1 was adjusted for age, sex; model 2 was adjusted for age, sex, marital status, race, education level, family income, smoking status, drinking status, hypertension, diabetes, stroke, coronary heart disease, BMI, energy consumption, protein consumption, carbohydrate consumption, fat consumption and C-reactive protein.

There was a significant negative association between dietary folate intake and severe headache, when analysed by quartiles of dietary folate intake, after adjusting for potential confounders. In crude, the unadjusted OR values for dietary folate intake and severe headache in Q2 (229·98–337 ug/d), Q3 (337·01–485 ug/d) and Q4 (≥ 485·01 ug/d), compared with individuals with lower dietary folate intake Q1 (≤ 229·97 ug/d), were 0·76 (95 % CI: 0·64, 0·90, *P* = 0·002), 0·82 (95 % CI: 0·70, 0·96, *P* = 0·013) and 0·57 (95 % CI: 0·49, 0·66, *P* < 0·0001), respectively (Table 3). In model 1, the adjusted OR values for dietary folate intake and severe headache in Q2 (229·98–337 ug/d), Q3 (337·01–485 ug/d) and Q4 (≥ 485·01 ug/d), compared with individuals with lower folate intake Q1 (≤ 229·97 ug/d), were 0·79 (95 % CI: 0·66, 0·94, *P* = 0·01), 0·92 (95 % CI: 0·78, 1·08, *P* = 0·29) and 0·66 (95 % CI: 0·56, 0·78, *P* < 0·0001), respectively (Table 3). In model 2, the adjusted OR values for dietary folate intake and severe headache in Q2 (229·98–337 ug/d), Q3 (337·01–485 ug/d) and Q4 (≥ 485·01 ug/d), compared with individuals with lower folate intake Q1 (≤ 229·97 ug/d), were 0·81 (95 % CI: 0·67, 0·98, *P* = 0·03), 0·93 (95 % CI: 0·77, 1·12, *P* = 0·41) and 0·63 (95 % CI: 0·49, 0·80, *P* < 0·001), respectively (Table 3).

After excluding the participants with extreme energy intake, 9605 participants remained, and the relationship between dietary folate intake and severe headache kept stable. Compared with individuals with lowest folate intake Q1 (≤ 231·81 ug/d), the adjusted OR values for dietary folate intake and severe headache in Q2 (231·82–336 ug/d), Q3 (336·01–480 ug/d) and Q4 (≥ 480·01 ug/d) were 0·84 (95 % CI: 0·68, 1·03, *P* = 0·09), 0·96 (95 % CI: 0·79, 1·18, *P* = 0·69) and 0·65 (95 % CI: 0·51, 0·84, *P* = 0·002 (Table 4), respectively (Table 4). In the restricted cubic spline analyses (online Supplementary Fig. S1), we found a non-linear association between folate intake and severe headache (*P* = 0·006). The OR values for the relationship between folate intake and severe headache were decreased with increasing folate intake.

**Table 4.** Association between dietary folate intake and severe headache in participants with extreme energy intake was not included

		Crude			Model 1			Model 2		
Quartiles	No.	OR	95 % CI	*P* value	OR	95 % CI	*P* value	OR	95 % CI	*P* value
Folate intake (ug/d)										
Q1(≤ 231·81)	2402	1·00 (reference)			1·00 (reference)			1·00 (reference)		
Q2(231·82–336)	2403	0·78	0·65, 0·94	0·01	0·82	0·67, 0·99	0·04	0·84	0·68, 1·03	0·09
Q3(336·01–480)	2406	0·85	0·72, 1·00	0·05	0·95	0·79, 1·13	0·54	0·96	0·79, 1·18	0·69
Q4(≥ 480·01)	2394	0·58	0·49, 0·69	< 0·0001	0·67	0·56, 0·81	< 0·0001	0·65	0·51, 0·84	0·002
*P* trend	–	< 0·0001		–	< 0·001		–	0·003		–

Crude was adjusted for nothing. Model 1 was adjusted for age and sex. Model 2 was adjusted for model 1 + marital status, race, education level, family income, smoking status, drinking status, hypertension, diabetes, stroke, coronary heart disease, BMI, energy consumption, protein consumption, carbohydrate consumption, fat consumption and C-reactive protein.

### Stratified analysis

**Fig. 2.**
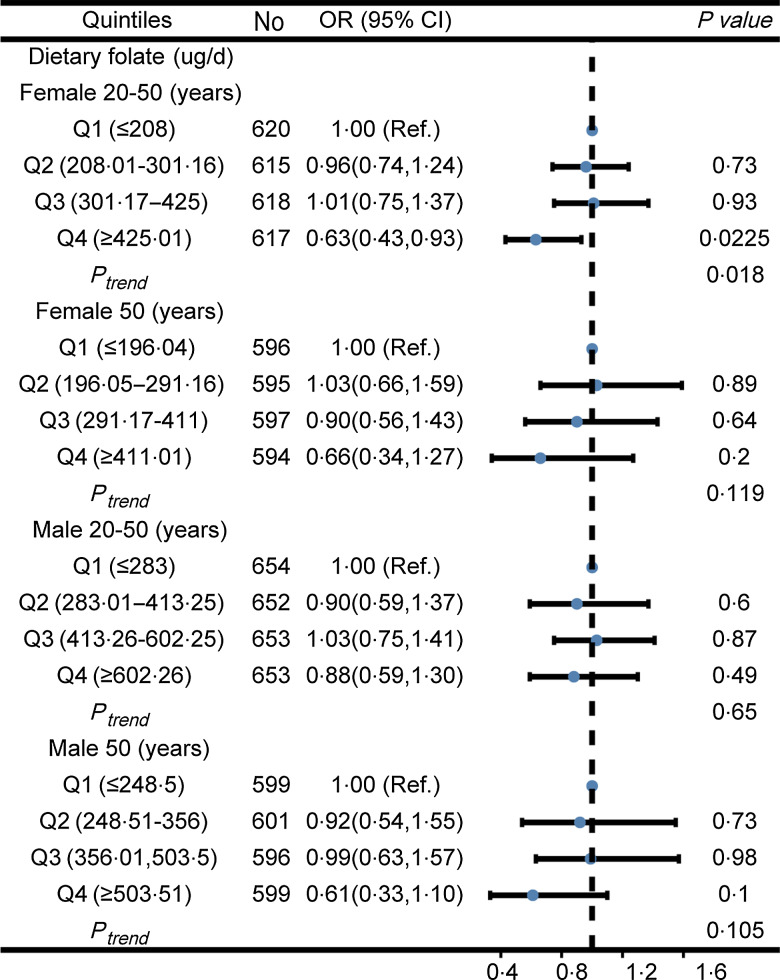
Association between dietary folate intake and severe headache in different sex and age groups. OR values were adjusted for marital status, race, education level, family income, smoking status, drinking status, hypertension, diabetes, stroke, coronary heart disease, BMI, energy consumption, protein consumption, carbohydrate consumption, fat consumption and C-reactive protein.

We found that dietary folate consumption was negatively associated with severe headache in women aged 20–50 years, stratified by age for both men and women. Compared with individuals with lower folate intake Q1, the adjusted OR values for dietary folate intake and severe headache in Q2, Q3, and Q4 were 0·96 (95 % CI: 0·74, 1·24, *P* = 0·73), 1·01 (95 % CI: 0·75, 1·37, *P* = 0·93) and 0·63 (95 % CI: 0·43, 0·93, *P* = 0·0225), respectively (Fig. 2). For women over 50 years of age and for adult men, there was no significant association between dietary folate consumption and severe headache (Fig. 2).

**Fig. 3.**
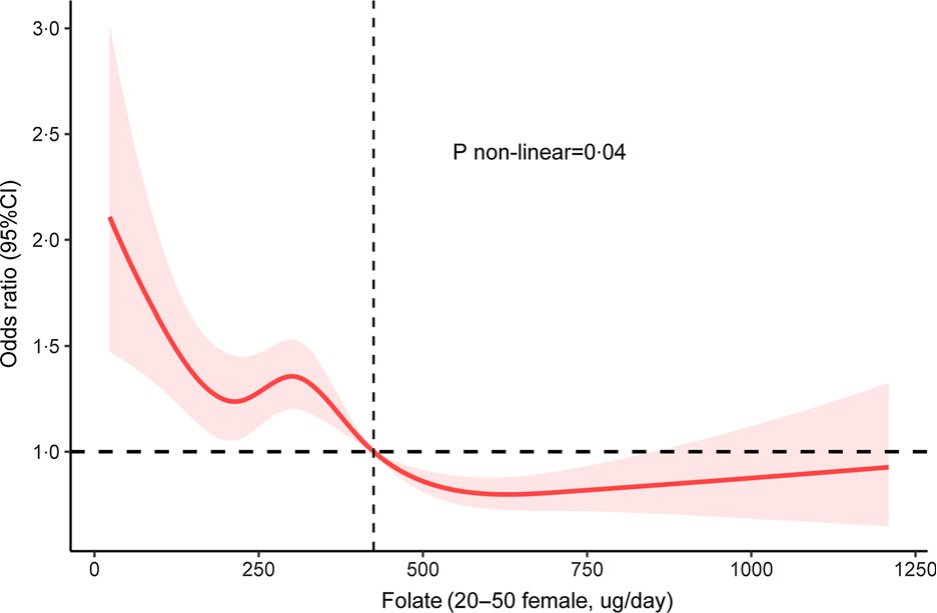
Association between dietary folate intake and severe headache in female of 20–50 years in RCS. The model was adjusted for marital status, race, education level, family income, smoking status, drinking status, hypertension, diabetes, stroke, coronary heart disease, BMI, energy consumption, protein consumption, carbohydrate consumption, fat consumption and C-reactive protein. Solid line, OR; shade, 95 % CI. RCS, restricted cubic spline

In the restricted cubic spline (Fig. 3), for women aged 20–50 years, we found a nonlinear relationship between dietary folate consumption (continuous variable) and severe headache (*P* = 0·04), using a reference point of 425 ug/d of dietary folate consumption. Moreover, we observed that the risk of developing severe headache declined with increasing dietary folate consumption until it reached 716 ug/d, after which the risk of severe headache reached a plateau.

## Discussion

In this large cross-sectional study, we provided the first nationally representative evidence of the relationship between dietary folate consumption and severe headache in American adults. Dietary folate intake was significantly and inversely associated with severe headache. The sensitivity analyses revealed a robust association between dietary folate intake and severe headache in adults. After stratified analyses by sex and age, we observed that dietary folate consumption was negatively associated with severe headache in women aged 20–50 years. For women over 50 years of age and for adult men, there was no significant association between dietary folate consumption and severe headache. And we further observed by restricted cubic spline analysis that the risk of developing severe headache decreases with increasing dietary folate consumption.

A great deal of interest has been accumulated in dietary interventions in the prevention of migraine and headache^(22)^. In 2015, Menon et al had already explored dietary folate intake and clarified its negative association with headache frequency^(23)^. There were also other nutrients that have been extensively investigated, among them vitamin B_2_ (riboflavin) and co-enzyme Q10 supplementation which have been shown to be beneficial for migraine patients^(19,24)^. Headache may be associated with hyperhomocysteinaemia^(25)^. In this context, homocysteine-lowering (Hcy) folate has been shown to be beneficial in the management and prevention of migraine^(26)^. It is noteworthy that most of the current studies are case reports or case series, and no specific studies have been conducted to explore the relationship between dietary folate and severe headache in the general population. The NHANES provides USA a unique opportunity to assess whether there is a negative relationship between dietary folate consumption and severe headache in the general population.

Although the underlying mechanism of the negative relationship between folate intake and severe headache is still to be investigated, our results are biologically plausible based on the available evidence. Currently, correlative applications of phosphorus nuclear magnetic resonance spectroscopy elucidate changes in energy metabolism in the brain of migraine patients^(27)^. It suggests that an imbalance between brain energy demand and ATP production plays an important role in severe headache. And since glycolysis is the main process of energy production in the brain, the energy-producing functions of mitochondria are closely related to the pathogenesis of migraine^(28)^. A study on family hemiplegic migraine revealed that the energy metabolism of brain and muscle in migraine patients was defective^(29)^. Additionally, a previous study showed that elevated levels of Hcy were related to mitochondrial dysfunction and energy production in the central nervous system^(30)^. However, the dietary compounds pyridoxine (vitamin B_6_) and folate have been identified to reduce Hcy levels^(31)^. All in all, folate deficiency may be related to headache attacks. These showed that maintaining normal mitochondrial function and reducing serum homocysteine properties of folate to may contribute to its beneficial effects on severe headache.

Severe headache or migraines are highly prevalent in adults under the age of 50 years and women, on average, are at roughly three times the risk of developing migraine than man^(32)^. In stratified analysis by gender and age, dietary folate consumption was only associated with severe headache in women aged 20–50 years old. Therefore, the results of this study further explain that dietary folate deficiency may be one of the reasons for the high prevalence of severe headache in women aged 20–50 years. As the body cannot synthesise folate, dietary intake is an important source of folate for the body, which is absorbed by the body in the proximal jejunum. Folate was found in many natural foods such as pulses, yeast, fruit and green leafy vegetables, especially dark green vegetables and in some animal foods such as liver and kidney^(33)^. Women, especially those of childbearing age, are becoming more aware of folate supplementation and are actively taking appropriate amounts of folate before and during pregnancy, and dietary folate intake is the preferred method of supplementation^(34–36)^. It is now mostly believed that the gastrointestinal tract is less functional in the elderly than in younger people^(37)^. As a result, older adults may have a reduced intake of dietary folate. Therefore, women aged 20–50 years may have higher folate absorption than men and women over 50 years, which may be the reason why dietary folate was associated with severe headache only in women aged 20–50 years. In the restricted cubic spline, when 425 ug/d of dietary folate consumption was used as a reference point, we observed that the OR was significantly lower than 1·00. Therefore, we recommend that women between the ages of 20 and 50 should have a daily dietary intake of the appropriate amount of folate. In sum, the negative association between folate intake and severe headache in a population of women aged 20–50 years is important for proposing strategies to prevent severe headache in adults in a specific population.

There are some limitations that must be taken into account in the study. At first, the diagnosis of severe headache or migraine was based on the self-report questionnaire ‘In the past 3 months, have you had severe headaches or migraines?’ In addition, no data was available on other characteristics of participants’ severe headaches or migraines, such as severity or other symptoms and which subtype of migraine. Therefore, we used the term severe headache uniformly. This may not be the best terminology, but at least it eliminates the confusion as if the headache was migraine or not. Second, dietary data were obtained through 24-h recall, an approach that had inherent limitations in terms of reliability and validity of nutritional assessment. However, Prentice RL *et al.* suggested that 24-h recall may provide more detail about food types and quantities than food frequency surveys^(38)^. Third, since the study was conducted on USA adults and did not include special groups such as minors, we cannot analyse special populations or other ethnicities which resulted in insufficient extrapolation power for this study. Therefore, further researches are necessary to verify the generalisability of these results. And, we cannot eliminate the interference of nonrandom missing data on the results because of baseline differences between included and excluded participants. Finally, our study was a cross-sectional study, which meant that causal inferences cannot be made. Thus, further prospective longitudinal investigations are needed to clarify the causal relationship between dietary folate intake and severe headache. Our study also has several advantages. This study provided epidemiological evidence of the significant relationship between dietary folate intake and severe headache in a representative general population across the USA. In addition, we provided reliable correlations by controlling for multiple potential confounders in our statistical analyses. Furthermore, we performed stratified analyses for both sex and age to detect differences between sex and age groups.

### Conclusions

Our study first explored the relationship between dietary folate consumption and severe headache in American adults. This study suggested that dietary folate may play an important role in the prevention of severe headache. We recommend that women aged 20–50 years should have higher awareness of folate and increase their dietary intake of folate if necessary.

## Supplementary material

For supplementary material accompanying this paper visit https://doi.org/10.1017/S000711452300137X. click here to view supplementary material
