# Supplementary material for: Association between dietary folate intake and severe headache among adults in the USA: a cross-sectional survey
Source: Br J Nutr. 2023 Jun 20;131(3):438–46. doi: 10.1017/S000711452300137X (PMC10784126; doi:10.1017/S000711452300137X)
Supplement: Supplementary file 1 [file S000711452300137Xsup001.docx]

**Table S1.** Basic characteristics of excluded and included participants

|  | Excluded population | Included population | *P* value |
| --- | --- | --- | --- |
| Number of subjects (%) | 21267(68.32%) | 9859 (31.67%) |  |
| Sex (%) |  |  | <0.001 |
| Male | 10177 (47.9) | 5007 (50.8) |  |
| Female | 11090 (52.1) | 4852 (49.2) |  |
| Age(year) | 19.90 (21.36) | 50.75 (18.47) | <0.001 |
| Race (%) |  |  |  |
| Non-Hispanic White | 6880 (32.4) | 5226 (53.0) | <0.001 |
| Non-Hispanic Black | 5780 (27.2) | 1792 (18.2) |  |
| Mexican American | 6573 (30.9) | 2115 (21.5) |  |
| Others | 2034 (9.6) | 726 (7.4) |  |
| Marital status (%) |  |  |  |
| Living alone | 7206 (70.5) | 3675 (37.3) | <0.001 |
| Married | 3017 (29.5) | 6184 (62.7) |  |
| Education (%) |  |  |  |
| <High school | 11712 (73.2) | 2978 (30.2) | <0.001 |
| High school | 1870 (11.7) | 2354 (23.9) |  |
| >High school | 2419 (15.1) | 4527 (45.9) |  |
| Family income (%) |  |  |  |
| Low | 8096 (44.0) | 2684 (27.2) | <0.001 |
| Medium | 6485 (35.2) | 3832 (38.9) |  |
| High | 3822 (20.8) | 3343 (33.9) |  |
| Smoking status |  |  |  |
| Never | 2961 (54.5) | 4944 (50.1) | <0.001 |
| Current | 1102 (20.3) | 2201 (22.3) |  |
| Former | 1375 (25.3) | 2714 (27.5) |  |
| Drinking |  |  |  |
| Never | 599 (18.6) | 1395 (14.1) | <0.001 |
| Current | 1907 (59.2) | 6421 (65.1) |  |
| Former | 715 (22.2) | 2043 (20.7) |  |
| Diabetes | 580 (2.9) | 996 (10.1) | <0.001 |
| Hypertension | 1749 (20.0) | 3265 (33.1) | <0.001 |
| Stroke | 284 (5.2) | 322 (3.3) | <0.001 |
| Coronary heart disease | 232 (4.3) | 475 (4.8) | 0.18 |
| BMI (kg/m^2^) | 22.78 (6.71) | 28.38 (6.21) | <0.001 |
| Energy (kcal/day) | 1978.27 (982.91) | 2121.86 (1023.94) | <0.001 |
| Protein intake (g/day) | 68.82 (39.83) | 79.70 (41.98) | <0.001 |
| Carbohydrate intake(g/day) | 264.03 (136.02) | 262.21 (134.40) | 0.29 |
| C-reactive protein (mg/dl) | 0.28 (0.70) | 0.47 (0.93) | <0.001 |
| Fat consumption (g/day) | 72.74 (42.04) | 79.03 (46.12) | <0.001 |
| Severe headache | 1080 (19.8) | 1965 (19.9) | 0.84 |

Data are presented as unweighted number (unweighted percentage) for categorical variables and unweighted means (SE) for continuous variables.
